# The Role of Non-Specific Interactions in Canonical and ALT-Associated PML-Bodies Formation and Dynamics

**DOI:** 10.3390/ijms22115821

**Published:** 2021-05-29

**Authors:** Alexander V. Fonin, Sergey A. Silonov, Olesya G. Shpironok, Iuliia A. Antifeeva, Alexey V. Petukhov, Anna E. Romanovich, Irina M. Kuznetsova, Vladimir N. Uversky, Konstantin K. Turoverov

**Affiliations:** 1 Laboratory of Structural Dynamics, Stability and Folding of Proteins, Institute of Cytology, Russian Academy of Sciences, 4 Tikhoretsky Ave., 194064 St. Petersburg, Russia; silonovsa25@yandex.ru (S.A.S.); olesyashpironok@gmail.com (O.G.S.); julgag@yandex.ru (I.A.A.); imk@incras.ru (I.M.K.); 2 Research Center for Molecular Mechanisms of Aging and Age-Related Diseases, Moscow Institute of Physics and Technology, 141700 Dolgoprudny, Russia; 3 Institute of Hematology, Almazov National Medical Research Centre, 197341 St. Petersburg, Russia; petukhov_av@almazovcentre.ru; 4 St-Petersburg State University Science Park, Resource Center of Molecular and Cell Technologies, Universitetskaya nab. 7-9, 199034 St. Petersburg, Russia; a.romanovich@spbu.ru; 5 Department of Molecular Medicine and Byrd Alzheimer’s Research Institute, Morsani College of Medicine, University of South Florida, Tampa, FL 33612, USA; 6 Peter the Great St.-Petersburg Polytechnic University, Polytechnicheskaya 29, 195251 St. Petersburg, Russia

**Keywords:** membrane-less organelles (MLOs), PML-bodies, promyelocytic leukemia protein (PML) isoforms, acute hydrogen peroxide-induced oxidative stress, fluorescence recovery after photobleaching (FRAP)

## Abstract

In this work, we put forward a hypothesis about the decisive role of multivalent nonspecific interactions in the early stages of PML body formation. Our analysis of the PML isoform sequences showed that some of the PML isoforms, primarily PML-II, are prone to phase separation due to their polyampholytic properties and the disordered structure of their C-terminal domains. The similarity of the charge properties of the C-terminal domains of PML-II and PML-VI isoforms made it possible for the first time to detect migration of PML-VI from PML bodies to the periphery of the cell nucleus, similar to the migration of PML-II isoforms. We found a population of “small” (area less than 1 µm^2^) spherical PML bodies with high dynamics of PML isoforms exchange with nucleoplasm and a low fraction of immobilized proteins, which indicates their liquid state properties. Such structures can act as “seeds” of functionally active PML bodies, providing the necessary concentration of PML isoforms for the formation of intermolecular disulfide bonds between PML monomers. FRAP analysis of larger bodies of toroidal topology showed the existence of an insoluble scaffold in their structure. The hypothesis about the role of nonspecific multiple weak interactions in the formation of PML bodies is further supported by the change in the composition of the scaffold proteins of PML bodies, but not their solidification, under conditions of induction of dimerization of PML isoforms under oxidative stress. Using the colocalization of ALT-associated PML bodies (APBs) with TRF1, we identified APBs and showed the difference in the dynamic properties of APBs and canonical PML bodies.

## 1. Introduction

For a very long time (starting from as early as the 17th century), biologists have observed cytoplasmic and nucleoplasmic bodies or compartments that did not have a lipid membrane. However, only 10–15 years ago it became clear that the formation and functioning of these cell compartments are based on the reversible liquid-liquid phase transitions of biopolymers in the cellular milieu [1,2]. Such compartments are called membrane-less organelles (MLOs) [2]. It was found that these organelles are dynamic liquid-droplet formations that constantly exchange their contents with the intracellular environment [3]. These organelles demonstrate the characteristic properties of liquids: wetting, surface tension, fusion upon contact with each other [4]. The composition of such organelles is largely represented by intrinsically disordered proteins [5,6,7,8] and ribonucleic acids—biopolymers characterized by high conformational flexibility [9,10]. The formation of such organelles occurs similarly to the concentration-dependent phase separation of polymer macromolecules in the polymer–solvent system [10,11]. It should be borne in mind that biopolymers function in a cell under conditions of macromolecular crowding, i.e., in conditions of limited free space. This significantly limits the possible conformational states of the biopolymers, primarily intrinsically disordered proteins, increases their local concentrations, and thereby facilitates the separation of biopolymers into phases [9]. The MLOs arising in this case should be considered as open, non-linear dynamic systems existing at the edge of chaos [12], which determines the essential dependence of their properties on small changes in the external conditions and the action of various stimuli.

Furthermore, an active component of all biological processes, including phase transitions of biopolymers of the “liquid-liquid” type, leading to the formation of membrane-less organelles, is water. The change in the structure of water in the presence of various solutes from small molecules to biological macromolecules can be the initial and decisive stage in the formation of membrane-less organelles [13].

PML-bodies are polyfunctional dynamic protein organelles present in most cell types, where they are involved in the regulation of transcription, cell differentiation, stress response, etc. [14,15]. As a rule, 5–30 PML bodies of predominantly toroidal topology with a diameter of 0.1–2 μm are observed in the cell nucleus. These structures typically do not contain nucleic acids. However, PML bodies are normally associated with chromatin. More than 170 proteins have been identified in PML bodies, many of which are involved in chromatin regulation [16].

The main protein of PML-bodies is the protein of promyelocytic leukemia (PML) [17]. PML received this name due to the discovery of the chimeric oncoprotein PML-RARα (expressed as a result of chromosomal translocation t (15; 17) (q22; q21) of the PML and the receptor of retinoic acid alpha genes) in 98% of cases of acute promyelocytic leukemia [15]. Due to alternative splicing, PML has at least seven main isoforms that differ in size and amino acid sequence of the C-terminal domain [18]. The N-terminal ordered region of the protein, which is the same in all PML isoforms, contains the so-called RBCC (Ring-Box-Coil-Coil) motif consisting of several zinc-binding RING (Really Interesting New Gene) domains and two B-domains (B->Boxes), and also the leucine-rich α-helical coil-coil domain [18]. The C-terminus of most PML isoforms contains a nuclear localization signal (NLS) and a SUMO-interacting motif (SIM). SIM and RBCC provide the possibility of specific interaction of PML with a large number of partners, and NLS promotes nuclear localization of PML-bodies [18]. The SIM motif plays an important role in the functional activity of PML—most of the proteins that make up PML bodies are sumoylated and are recruited into these organelles due to SUMO/SIM interactions. The PML-VII isoform (does not contain NLS) and the PML-I isoform (contains the nuclear export signal, NES) can be localized not only in the nucleoplasm but also in the cytoplasm [19]. PML isoforms differ significantly in the length of the amino acid sequence and its composition, charge, and degree of ordering of the protein structure. Nuclear forms of PML can be localized in different regions of the nucleus [19]. Post-translational modifications of this protein, especially sumoylation, play an essential role in the functioning of PML. The N-terminal domain of PML undergoes this modification at sites K65, K160, K226, K490, which allows molecules of different PML isoforms to oligomerize and attract other proteins (Sp100, DAXX) via SUMO/SIM interactions.

In addition to the canonical PML-bodies, the PML protein is able to form alternative structures, usually of pathological nature. For example, in immunodeficiency, centromeric region instability, facial anomalies syndrome (ICF), and several other diseases, giant toroidal PML bodies are formed, containing, in addition to the main PML body proteins, uncoded heterochromatin. Infection with the herpes simplex virus HSV-1 leads to the formation in the host cell of viral DNA-containing PML nuclear bodies (vDCP NBs), PML-bodies containing the double-stranded DNA of the herpes virus. Of particular interest are PML-bodies associated with alternative telomere lengthening, ALT-associated PML-bodies (APBs). APBs are observed in approximately 90% of ALT-positive cells, are involved in telomere lengthening, serve as markers of the “canonical” ALT mechanism, and are considered a potential therapeutic target. These bodies are formed by the PML protein and include proteins Sp100, SUMO-1/2/3, proteins involved in DNA Damage Response (DDR), DNA repair, recombination, replication and chromatin organization, as well as telomeric DNA and telomere-binding proteins [20,21,22,23]. During the formation of APBs, the proteins that make up shelterin (a six-subunit protein complex containing TRF1, TRF2, POT1, TPP1, TIN2, and Rap1 and specifically associating with mammalian telomeres) are separated from telomeric DNA and incorporated into APBs, which creates conditions for homologous recombination and repair synthesis of unprotected telomeric DNA and non-homologous end joining (NHEJ) [21,22,23,24]. Telomere-binding proteins TRF1 and TRF2 play a special role in the formation and functioning of APBs [21,23,24,25]. Dissociation of TRF2 and telomeric DNA during the formation of APBs causes activation of ATM kinase (ataxia telangiectasia mutated), a key regulator of DDR and DSB signaling pathway that triggers the process of telomeric DNA repair by its homologous recombination in APBs [21]. ATM kinase also promotes TRF1 migration to APBs [24]. This protein directly interacts with some isoforms of the scaffold protein APBs–PML, possibly facilitating the incorporation of telomeric DNA into APBs [26,27,28]. In this case, the phosphorylation and sumoylation of TRF1 play an essential role in the formation of functionally active APBs [24,25,28]. According to modern concepts, the formation of APBs is due to the interaction of pre-assembled PML-based structures with telomeres [21,22]. This interaction triggers a cascade of post-translational modifications of the PML protein and shelterin proteins, which promotes the compaction of telomeric DNA [20,21,22]. The most significant contribution to the formation of functionally active APBs is made by the sumoylation of shelterin and PML proteins [20,21,22]. This facilitates the interaction of PML with another APB scaffold protein, Sp100, and the incorporation of a number of DDR and DSB factors into APBs through the interaction between modified proteins of forming bodies and SIM motifs of proteins involved in DDR and DSB [20,21,22]. It is assumed that the scaffold envelope of functionally active APBs is formed by sumoylated proteins PML and Sp100, and telomeric DNA and ECTR, DDR and DSB factors, and several shelterin proteins, including TRF1, are localized in the internal cavity of APBs, where telomeric DNA is lengthened [20,21,22,29].

In view of the exceptional biological significance of PML bodies for many cellular processes in health and disease, the study of these compartments began long before the emergence of fundamentally new ideas about the role of weak intermolecular nonspecific interactions and phase transitions of the liquid-liquid type in the formation of membrane-less organelles.

The purpose of this work was to draw the attention of researchers studying PML bodies to the question that neither the formation of S-S bonds nor SUMO/SIM interactions can be the primary cause of PML-bodies formation; to prove that polypeptide sequences of PML isoforms are prone to liquid-droplet (membrane-less organelles) formation; and to study the dynamic properties of canonical and ALT-associated PML-bodies and the effect of oxidative stress on them. Results of this study show that weak interactions, rather than strong interactions, play a decisive role in the formation of these membrane-less organelles even under conditions favorable for disulfide bond formation and SUMO/SIM-contacts between PML molecules.

## 2. Results and Discussion

In this work, based on the analysis of the amino acid sequences of PML isoforms, it was hypothesized that nonspecific interactions play a decisive role in the transition of PML isoforms to the condensed state as the initial stage of PML body formation. This hypothesis was validated by the FRAP-based characterization of the canonical and ALT-associated PML-bodies and the analysis of their liquid-droplet properties under normal conditions and under the conditions of oxidative stress that promoted oxidative dimerization of PML monomers.

### 2.1. Analysis of Amino Acid Sequence of PML Isoforms

The domain structure of PML isoforms is the result of alternative mRNA splicing. The N-terminal RBCC domain, which is the same for all PML isoforms (amino acid residues 1–394), is encoded by the first three exons of the gene of this protein, and, according to the data of X-ray structural analysis [30,31,32], has an ordered structure, which is consistent with the results of our analysis of the PML intrinsic disorder predisposition (Supplementary Figure S1). Exons 4–9 participate in the formation of the rest of the amino acid sequences of PML isoforms. Amino acid residues 395–552, encoded by exons 4, 5, and 6 of the *PML* gene, are the same for the nuclear isoforms PML-I–PML-VI. The isoforms PML-I–PML-V also have the same amino acid residues 552 to 570, which contain the SIM motif and are encoded by the common region of the seventh exon for these forms. The PML-IV isoform contains a unique region of the amino acid sequence encoded by a portion of the eighth exon 8b, which is not found in other PML isoforms. The C-terminal variable domains of PML isoforms formed as a result of alternative splicing of the primary PML transcript usually include regions of the PML sequence encoded by a combination of different regions of exons 7–9, i.e., regions of the PML sequence following the amino acid residue 552. The role of residues 394 to 552, encoded by 4–6 exons of the PML gene, in the structure of PML isoforms is often not discussed at all, since this region of the amino acid sequence does not belong either to the RBCC motif or to the C-terminal domains of PML isoforms. In this case, the fifth and sixth exons are not involved in the formation of the cytoplasmic isoform PML-VII. DNA of one of the variants of the chimeric oncoprotein PML-RARα is formed as a result of the fusion of the first three exons of the PML gene and the gene for the retinoic acid receptor type alpha [33]. Thus, the regions of the amino acid sequence of PML from 394 to 552 a.a. are also variable in different forms of this protein.

**Figure 1:**
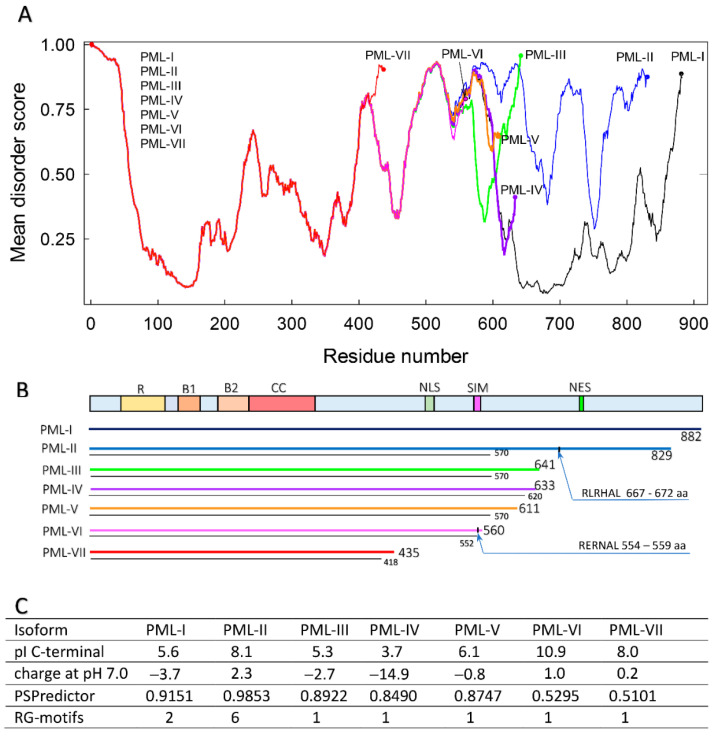
Schematic presentation of different alternatively spliced isoforms of human PML protein. (**A**) Mean disorder score. Disorder score determined by s(MDP), IUP2(S), IUP2(L), VLXT, VL3, VSL2B, PONDR-FIT algorithms are presented in Figure 1. (**B**) The generalized domain structure, indicating positions of the Ring domain (R—amino-acid residues 45–105), Box 1 (B1—residues 124–166), Box 2 (B2—residues 184–230), Coil-coil (CC—residues 229–323), Positions of the nuclear localization signal (NLS—residues 476–490), SUMO-interacting motif (SIM—residues 556–562) and nuclear export signal (NES—residues ~704–713, exclusive to isoform I). Double lines show the length of each isoform (colored line) and the length of the fragment in which the amino acid residues are the same in PML-I isoforms. Arrows indicate the position of membrane binding domains (667–672 a.a. in PML-II and 554 a.a. in PML-VI). (**C**) Different characteristics of PML-isoforms: pI of C-terminal domain, the charge of protein at pH 7.0, the result of LLPS prediction analysis, the content of RG-motifs in amino acid sequence.

Analysis of the amino acid sequences of all PML isoforms showed that the structure of these proteins, determined by the sequences encoded in the PML gene by exons 4–9, is significantly disordered (Figure 1).

In our opinion, these data also make it possible to assign the amino acid sequence regions of PML isoforms, encoded by exons 4–6, to the C-terminal domains of these proteins, formed as a result of alternative splicing of the primary PML transcript.

According to the currently accepted model of PML body formation [14,33,34,35], oxidative dimerization of PML isoforms due to the formation of disulfide bonds between monomers of this protein initiates the formation of an insoluble “aggregate” to which, due to SUMO/SIM interactions, client proteins are attached. Interactions in the region of the RBCC motif play an essential role in the formation of functionally active PML bodies and the inclusion of at least the PML-I isoform in their composition. According to [19], the replacement of conserved cysteine residues 57 and 60 with serine residues results in a uniform distribution of this isoform in the nucleus but does not change the localization of PML-II, which remains in the PML-bodies. It has been shown in vitro that the N-terminal domains of PML are capable of forming tetramers and higher-order complexes [30,31]. The formation of such structures critically depends not only on the formation of disulfide bonds but also on hydrophobic and electrostatic interactions determined by conserved residues localized in the B1 and RING domains of the RBCC motif [30,31].

However, oxidative dimerization of PML requires a high concentration of PML molecules and enzymes that catalyze the formation of disulfide bonds “in the right place at the right time”. Interactions in the region of conserved residues of the RBCC motif also require a high protein concentration. Accordingly, for the de novo formation of PML bodies, PML pre-condensation is required. In our opinion, this can occur as a result of liquid-liquid phase separation of PML isoforms, which appears due to multiple weak nonspecific interactions of intrinsically disordered regions of PML isoforms.

The role of liquid-liquid phase transitions in the formation of PML bodies has been discussed in several studies [36,37,38,39,40,41]. According to their authors, LLPS of PML isoforms is due to multivalent specific SUMO/SIM interactions and is observed at the late stages of PML body formation. Indeed, to participate in SUMO/SIM interactions, the PML polypeptide chain must be subjected to polysumoylation, phosphorylation in the region of the SIM motif, and other post-translational modifications, which is hardly possible under conditions of a uniform distribution of PML in the nucleus [32,42,43,44]. The need for preliminary oligomerization of PML to attract to these structures the key enzyme UBC-9 for PML sumoylation was shown in [30].

When analyzing the literature data, we found that the results of some studies [19,45,46] can be interpreted in the context of LLPS of PML isoforms, which is determined by the intrinsic properties of the amino acid sequences of these proteins and does not depend on their post-translational modifications. For example, it was shown in [45] that the expression of PML-II and PML-VI isoforms under endogenous PML knockout in HeLa cells (PML^-/-^) induces the formation of spherical structures with a uniformly distributed protein in them, rather than toroidal bodies. Spherical morphology is characteristic of biomolecular condensates formed as a result of a liquid-liquid phase transition [4]. In the same work, it was shown that the toroidal morphology of PML bodies is due to the presence of a SIM motif in the sequence of PML isoforms and is determined by SUMO/SIM interactions [45]. In this case, the formation of spherical morphology bodies by the PML-II isoform does not depend on the presence of a SIM motif in the sequence of this protein and the sumoylation of the protein. At the same time, according to the data of [46], the PML-II isoform is incorporated into PML bodies independently of the N-terminal domain of this protein. These data indicate the ability of at least the PML-II isoform to be incorporated into PML-bodies, regardless of the formation of intermolecular disulfide bonds and SUMO/SIM interactions.

We analyzed the amino acid sequences of PML isoforms for the susceptibility to liquid-liquid phase separation. It is known that amino acid sequences with a high degree of disorder, possessing polyampholytic properties, containing tandem repeats RG, FY, FG, SY, SG, characteristic of sequences with a low degree of complexity, are prone to phase transitions of the “liquid-liquid” type [1,4,47]. Such sequences provide the possibility of multiple nonspecific electrostatic, cation-π and π-π weak intra and intermolecular interactions, which cause a decrease in the free energy of the polymer/solvent system necessary for the separation of biopolymers into phases [48,49]. The transition of intrinsically disordered proteins to the liquid-droplet phase can be caused both by a combination of such interactions of different physical nature and by some of their types, for example, electrostatic [50]. In this case, the contribution of one type or another of interactions to the phase separation of IDPs can vary depending on the properties of the solvent [13,51]. Proline-rich sequences are also characteristic of some IDPs that undergo phase separation [52]. Our analysis of the amino acid sequences of PML isoforms showed that the variable C-terminal domains of almost all PML isoforms have properties characteristic of sequences potentially capable of liquid-liquid phase separation due to electrostatic interactions. The C-terminal regions of PML isoforms are highly disordered, contain a large number of charged residues, and tandem repeats of the SS and PP type.

We also analyzed PML isoforms for susceptibility to phase separation using the PSPredictor software package [53] based on machine learning algorithms. This analysis showed that the amino acid sequences of all the studied isoforms are characteristic of proteins capable of LLPS. According to these data, the isoforms PML-II and PML-I have the highest probability of participating in the liquid-liquid phase separation. The results of the analysis of the amino acid sequence of the PML-II isoform for susceptibility to phase transitions of the liquid-liquid type using the PSPredictor are quite predictable. The C-terminus of the PML-II isoform contains the highest proportion of disordered regions of all the proteins studied. In addition, only in the C-terminal domain of PML-II, there are numerous RG motifs characteristic of sequences with a low degree of complexity, which determine the ability to phase separation of biopolymers due to cation-π and π-π weak interactions. Together with the available literature data on RBCC-free and SUMO/SIM-free incorporation of this protein into PML bodies [19,45,46], this allows us to conclude that the PML-II isoform can form liquid-droplet-like structures regardless of the interaction with other proteins.

The predicted by PSPredictor high propensity of the PML-I isoform to undergo liquid-liquid phase transitions is apparently due to the presence in the amino acid sequence of this protein of two RG and two YS motifs characteristic of ID proteins participating in LLPS. The high proportion of ordered regions in the amino acid sequence of the C-terminal domain of the PML-I isoform and the literature data on the critical importance of interactions in the RBCC-motif [19,30] for the incorporation of this protein into PML bodies indicate that this isoform is less prone than other PML isoforms to phase separation without interacting with their partners.

### 2.2. Sizes, Morphology, and Localization of PML-Bodies

**Figure 2:**
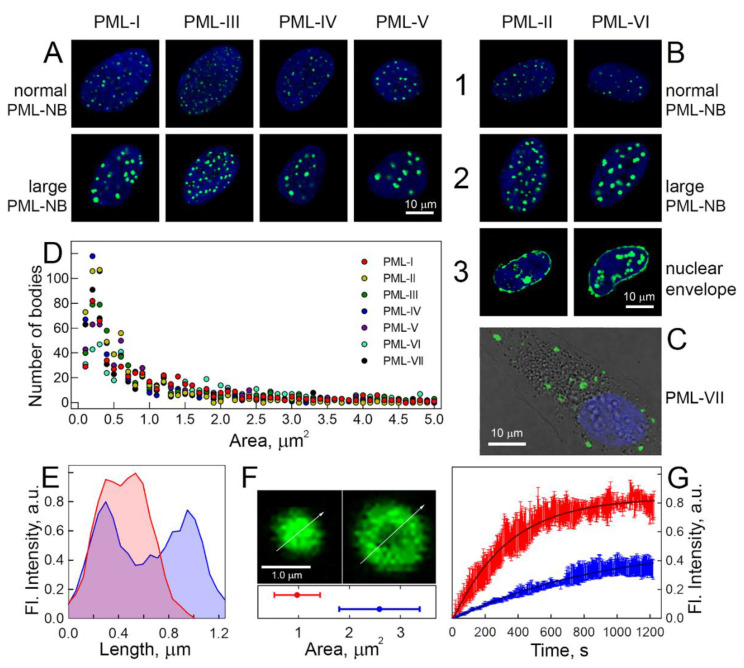
Localization, shape, size distribution, and dynamic properties of PML bodies visualized by various exogenous EGFP-PML isoforms in the presence of all endogenous forms of PML protein in U2OS cells. Nuclear localization of normal, small (area < 1.5 μm^2^, row 1) and large (area > 1.75 μm^2^, row 2) PML-bodies with exogenous PML isoforms I, III, IV, and V (**A**) and PML isoforms II and VI (**B**). Row 3 (**B**)—Migration of exogenous PML-II and PML-VI to the nuclear lamina. (**C**) cytoplasmic localization of PML bodies visualized by exogenous GFP-PML VII. (**D**) Distribution of different PML bodies by size. In all figures representing images of PML bodies, the scale bar is 10 μm. The nuclei are colored with DAPI. (**E**) Radial distribution of EGFP fluorescence intensity for small spherical (<1.5 μm^2^; red) and large toroidal (>1.75 μm^2^; blue) PML-bodies. (**F**) Micrographs of small spherical and large toroidal PML-bodies. The red and blue lines at the bottom show the body sizes measured by the EGFP fluorescence recovery curves (FRAP) shown in (**G**). (**G**) FRAP curves after photobleaching of nuclear PML isoforms in PML bodies. Photoreduction curves of PML isoforms in small and large PML-bodies are shown in red and blue. The approximation of FRAP data within the mono-exponential approximation is shown by solid black curves. The initial fluorescence for all bodies before photobleaching is taken as unity.

The exogenous expression of the chimeric EGFP-PML-I-VII proteins in the presence of endogenous PML was visualized using the confocal fluorescence microscopy analysis of the studied cells (Figure 2).

It was found that the chimeric EGFP-PML-I-VI proteins are predominantly localized in the nucleoplasm, whereas the EGFP-PML-VII isoform is predominantly distributed in the cytoplasm of the U2OS cells. Nuclear isoforms PML-I-VI in the nucleoplasm of U2OS cells were partially diffused throughout the entire nucleoplasm, partially concentrated in structures, the size and morphology of which were characteristic of PML-bodies. In the nucleoplasm of U2OS cells, the existence of such bodies with different sizes and morphologies was established, with all PML isoforms being involved in their formation. The results obtained are consistent with the literature data on the localization and morphology of PML- bodies in the nucleoplasm of U2OS cells [19,29,54,55]. The PML-VII isoform in the cytoplasm of U2OS cells also forms body-like structures. It is known that similar bodies are formed by mutant forms of PML with cytoplasmic localization, which accumulate in early endosomes [19]. When analyzing the expression of the EGFP-PML-II isoform in the nucleoplasm of U2OS cells, we found cells, in which the localization of the PML-II isoform is redistributed from the PML-bodies to the nuclear membrane. It is known that this protein could bind to the nuclear lamina and destroy it, which allows PML-II to partially move into the cytoplasm of U2OS cells [19,56]. The migration from PML-bodies to nuclear lamina was shown for EGFP-PML-VI protein as well.

It has been found that the distribution, size, and morphology of PML-bodies are not uniform. In U2OS cells, fluorescent bodies of spherical, toroidal, and cylindrical topology were observed. It was shown that the distribution of bodies by size and morphology does not depend on the visualized PML isoform. The exception is the compartments formed by the PML-VII isoform in the cytoplasm of the studied cells, as these formations represent larger structures in comparison with the nuclear PML bodies.

It is known that all nuclear PML isoforms are involved in the formation of PML-bodies [20]. The N-terminal domain of all PML isoforms and the C-terminal domains of PML-I, III, IV, V, and VII isoforms are negatively charged, while the C-terminal domains of PML-II and PML-VI isoforms bear a significant positive charge, which sharply distinguishes them from negatively charged C-terminal regions of other PML isoforms. Accordingly, it can be expected that potential multiple weak electrostatic interactions (polyelectrostatic interactions) can arise both between oppositely charged PML isoforms and between PML-II and PML-VI protein monomers. It should be taken into account that not only the complex coacervation of oppositely charged polymers [57] but also their change in the structure of water [13] can be the driving force of LLPS.

The positive charge of the C-terminal regions of the PML-II and PML-VI isoforms suggested the same functional activity of these proteins. In fact, the PML-II isoform in U2OS cells is capable of migrating from PML bodies to the inner membrane of the cell nucleus [56]. Considering that the nuclear membrane is negatively charged, electrostatic interactions may underlie such a redistribution of this protein in the nucleoplasm of U2OS cells. Then, the PML-VI isoform should have the same properties.

In order to confirm this hypothesis, we analyzed the localization of all PML isoforms in U2OS cells. We found cells with a similar redistribution of protein localization from PML-bodies to the nuclear membrane in the U2OS nucleoplasm upon expression of the EGFP-PML-VI isoform as well. To the best of our knowledge, this effect of relocalization from PMLs to the membrane is shown for the PML-VI isoform for the first time. The amino acid sequence of the C-terminal region of the PML-VI isoform consists of only eight amino acid residues GRERNALW. Six amino acid residues of this sequence (553–559 aa) RERNAL show 67% sequence identity to the RLRHAL motif (667–672 aa) found in the C-terminal region of PML-II, which is responsible for the migration of this protein to the nuclear periphery [19]. Both of these motifs are positively charged sequences. Therefore, given the negative charge of the intranuclear membrane, it can be assumed that the migration of PML-II and PML-VI isoforms to the periphery of the cell nucleus is due to electrostatic interactions.

### 2.3. PML-Bodies Associated with Alternative Telomere Lengthening

**Figure 3:**
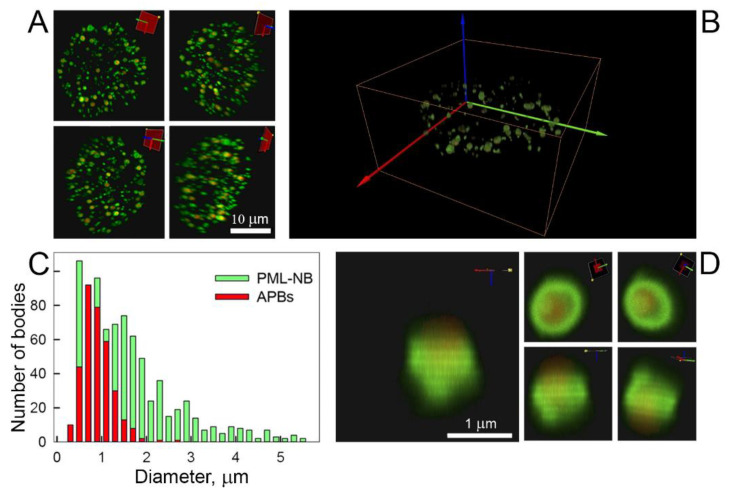
PML-bodies associated with alternative telomere lengthening (APBs). (**A**) Image of APBs with co-expressed by EGFP-PML-III and TagRFP-TRF1 using confocal fluorescence microscopy. Presented by APB in four angles. (**B**) 3D-structure of APBs with co-expressed by EGFP-PML-III and TagRFP-TRF1 using confocal fluorescence microscopy (see Video in Figure S2, Panel B). (**C**) Size distribution of PML-bodies colocalized with TRF1 in U2OS cells. (**D**) Visualization using fluorescence confocal microscopy of EGFP and TagRFP of the distribution of PML and TRF1 with coexpression of EGFP-PML-III and TagRFP-TRFP1 in U2OS cells (see Video in Figure S2, Panel B).

In addition to the normal PML bodies characteristic of all cell types, ALT-positive cells, which include U2OS cells, exhibit abnormal PML bodies associated with alternative telomere lengthening (APBs). It is known that telomeric DNA, extrachromosomal DNA, telomere-binding proteins, and shelterin proteins, including TRF1, are localized inside these bodies [21]. TRF1 plays an essential role in the formation of APBs, and it is believed that sumoylation of this protein initiates the formation of such bodies [22]. Therefore, for in vivo visualization of pathological PML bodies associated with alternative lengthening of telomeres in U2OS cells, cotransfection of constructs encoding chimeric EGFP fusion proteins with various PML isoforms and a construct encoding the TRF1 fusion protein with the red fluorescent protein TagRFP was carried out. Bodies colocalized with TRF1 were considered as APBs in U2OS cells. It was found that all PML forms, except for the cytoplasmic PML-VII, are involved in the formation of PML bodies colocalized with TRF1 (Figure 3).

These bodies represent a fairly uniform population in size and morphology. These are organelles of medium size (about 1 μm) of exclusively toroidal topology, within the ring-shaped structure of which TRF1 is localized. These results are consistent with the literature data on the size and morphology of APBs obtained from the analysis of colocalization of these bodies, telomeric DNA, and shelterin proteins in fixed cells [22,29]. Control experiments in ALT-negative cells showed no colocalization of PML isoforms with TRF1. Therefore, in the population of PML bodies, it was possible to isolate bodies associated with alternative lengthening of telomeres from the entire pool of PML-bodies in living U2OS cells.

Contransfection of PML isoforms with the shelterin protein TRF1 made it possible to isolate from the entire pool of PML bodies in U2OS cells a fairly uniform population of the ALT-associated PML bodies with a toroidal shape. PML isoforms in such bodies are rather slowly exchanged with nucleoplasm, and PML-V isoform is practically immobilized in these organelles.

### 2.4. Dynamics of Exchange of PML Isoforms in PML-Bodies with Nucleoplasm

The recovery of fluorescence after photobleaching (FRAP) is actively used to study the biophysical properties of droplets formed as a result of the phase separation of biopolymers both in vitro and in cellulo [58]. The structures that have liquid-droplet properties are distinguished by the rapid dynamics of the exchange of bleached and unbleached molecules and the almost complete restoration of fluorescence after its burning out.

**Figure 4:**
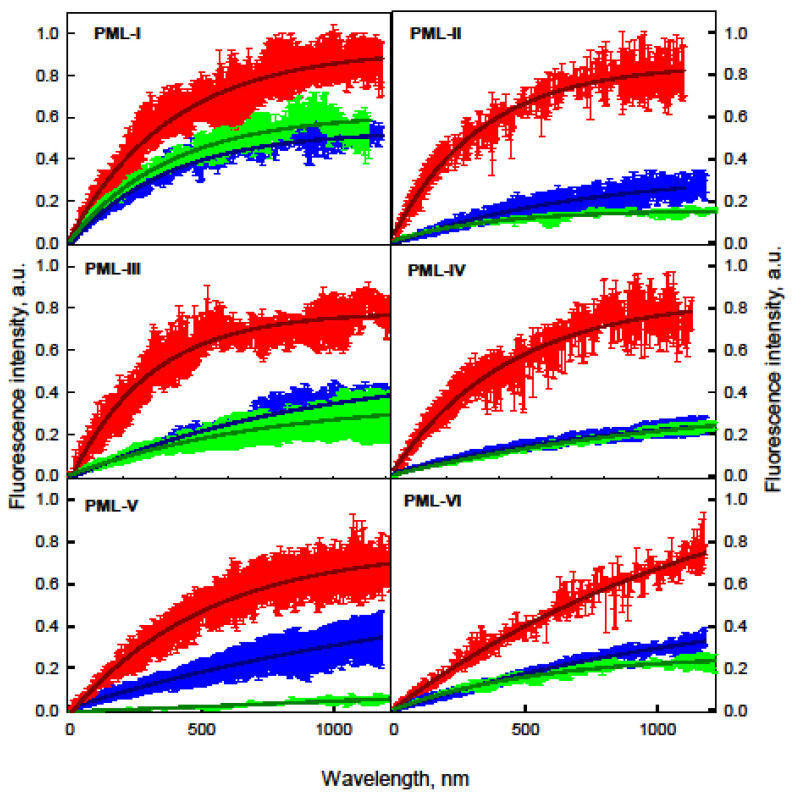
Curves of EGFP fluorescence recovery after photobleaching of nuclear PML isoforms in PML-bodies in U2OS cells. The curves of the photoreduction of PML isoforms in the composition of “small” PML bodies are shown in red, the curves of the photoreduction of PML isoforms in the composition of “large” PML bodies are shown in blue, and the curves of the photoreduction of PML isoforms in the composition of APBs are shown in green. Solid curves represent the approximation of FRAP data in the framework of the mono-exponential approximation. The standard deviation of the data is passed by error bars of the corresponding color.

Analysis of the distribution of PML bodies in U2OS cells showed the existence of small (less than 1 μm) bodies with a spherical topology and larger membrane-less organelles, a significant part of which is represented by toroidal bodies (Figure 4).

It was found that the heterogeneity of the size and topology of PML-bodies in U2OS cells correlated with the presence of several populations of PML-bodies with different characteristics of the EGFP fluorescence recovery after photobleaching of chimeric EGFP-PML-I-VI proteins. Analysis of FRAP characteristics allowed conventionally subdivide PML-bodies into two groups: “small” (area less than 1 μm^2^) and “large” (area more than 1 μm^2^). The lowest exchange dynamics with the nucleoplasm among all the PML isoforms included in the population of “large” nuclear PML-bodies was in the PML-V isoform. According to the literature, this isoform is the most stable component of PML-bodies [55,59].

PML isoforms that are part of the “small” bodies (including the PML-V isoform) are much more rapidly exchanged with the nucleoplasm of the cell in comparison with the proteins that are part of the “large” bodies. In this case, the proportion of the mobile fraction of PML isoforms included in the spherical bodies is significantly higher than the proportion of the mobile fraction of these proteins included in the “large” bodies. These results allow us to conclude that “small” bodies are more “liquid” compartments compared to the “large” PML bodies.

We also characterized the dynamics of cytoplasmic formations based on the PML-VII isoform, which are characterized by a slow recovery of EGFP-PML-VII fluorescence after photobleaching of this protein. The proportion of the mobile fraction EGFP-PML-VII in such bodies does not exceed 25% (Supplementary Figure S3).

Analysis of EGFP fluorescence recovery after photobleaching of chimeric EGFP-PML-I-VI proteins within APBs showed that the dynamics of the exchange of PML isoforms within the APBs corresponds to the dynamics of the exchange of “large” PML bodies with nucleoplasm, with the exception of the PML-V form. In the case of APBs, this PML form practically does not exchange with the cell nucleoplasm.

PML bodies have a property characteristic of liquid coacervates; i.e., the ability to exchange molecules with the environment. At the same time, we found a correlation between the characteristics of EGFP fluorescence recovery after photobleaching of chimeric proteins EGFP-PML-I-VI, which are part of the PML-bodies, and the morphology and size of these membrane-less organelles. We found the existence of a population of “small” PML bodies that do not have a pronounced toroidal structure, whose properties, according to FRAP analysis, are close to those of liquid-droplet compartments. At the same time, high dynamics of the exchange of proteins that make up the population of such bodies with nucleoplasm were found, even for the PML-V isoform, the most stable component of mature PML bodies. FRAP-analysis of PML isoforms included into the larger bodies of toroidal topology showed the existence of an insoluble scaffold with a high proportion of immobilized proteins in the structure of such bodies. In light of the performed analysis of the amino acid sequences of PML isoforms, small highly dynamic bodies can be considered as the PML bodies formed as a result of the liquid-liquid phase transition of PML isoforms. The maturation of these condensates into toroidal functional PML bodies is apparently due to the subsequent PML-PML interactions (including the formation of intermolecular disulfide bonds) and the attraction of client proteins by the SUMO/SIM mechanism [45].

FRAP analysis showed low dynamics of the structures formed by the PML-VII isoform. This isoform with cytoplasmic localization is actually an ordered N-terminal domain of PML, which contains practically no disordered regions and, therefore, is not capable of LLPS. It is known that structures based on PML-VII are localized in late endosomes [60]. The substitutions C57S and C60S, as well as for PML-I, cause diffuse distribution of PML-VII [19]. Taken together, this allows us to conclude that for PML oligomerization, due to the formation of disulfide bonds between the monomers of this protein, PML condensation is required—either in the “embryos” of PML bodies in the nucleus or in specific compartments in the cytoplasm.

It is known that sumoylation of shelterin proteins, which ensure their interaction with PML proteins by the SUMO/SIM mechanism, plays a significant role in the formation of pathological ABPs [22,37]. The low dynamics of the exchange of PML isoforms in APBs and the exclusively toroidal structure of such bodies can be considered as further evidence of the need for SUMO/SIM interactions for ordering the structure of PML bodies. The difference between the dynamic structure of APBs and the canonical PML bodies may underlie the pathological functional activity of ALT-associated PML-bodies.

### 2.5. The Influence of Acute Oxidative Stress on the Characteristics of the Exchange of PML Isoforms in PML-Bodies with the Environment

Within the existing model of PML-body formation, oxidative stress should induce solidification of PML-bodies due to the induction of the formation of disulfide bonds between PML monomers with an increase in the concentration of reactive oxygen species in cells. Moreover, oxidative stress could alter the profile of post-translational PML modifications, at least in the K487 (acetylation)/K490 (sumoylation) region [61]. These modifications are mutually exclusive. Deacetylation of K487 and, accordingly, an increase in PML sumoylation is observed under conditions of H_2_O_2_-induced oxidative stress [61]. In [62], a decrease in the dynamics of PML-bodies in response to oxidative stress caused by As_2_O_3_ was shown. However, treatment of cells with arsenic compounds causes dissociation of the PML-I, PML-II, and PML-VI isoforms from the PML-bodies, which is explained by the desumoylation of PML. At the same time, it is stated in [34] that As_2_O_3_, on the contrary, enhances PML sumoylation by attracting the RING-type E3 SUMO transferase UBC9. It is also known that arsenic trioxide directly binds to PML [63].

Therefore, we decided to investigate the effect of oxidative stress on the properties of PML bodies under the conditions of treatment of the studied cells with H_2_O_2_, which acts on proteins indirectly. Based on the assumption that the greatest changes in the properties of PML-bodies will be caused by the change of protein-protein interactions, the MTT test was used to determine the threshold H_2_O_2_ concentrations that did not lead to the cell death. Oxidative stress in U2OS cells was induced by treatment with 500 μM H_2_O_2_. The treatment of the studied cells with H_2_O_2_ had practically no effect on the morphology and localization of PML-bodies. At the same time, stress conditions had a different effect on the characteristics of the exchange of isoforms of EGFP-PML-I-VI proteins with nucleoplasm.

**Figure 5:**
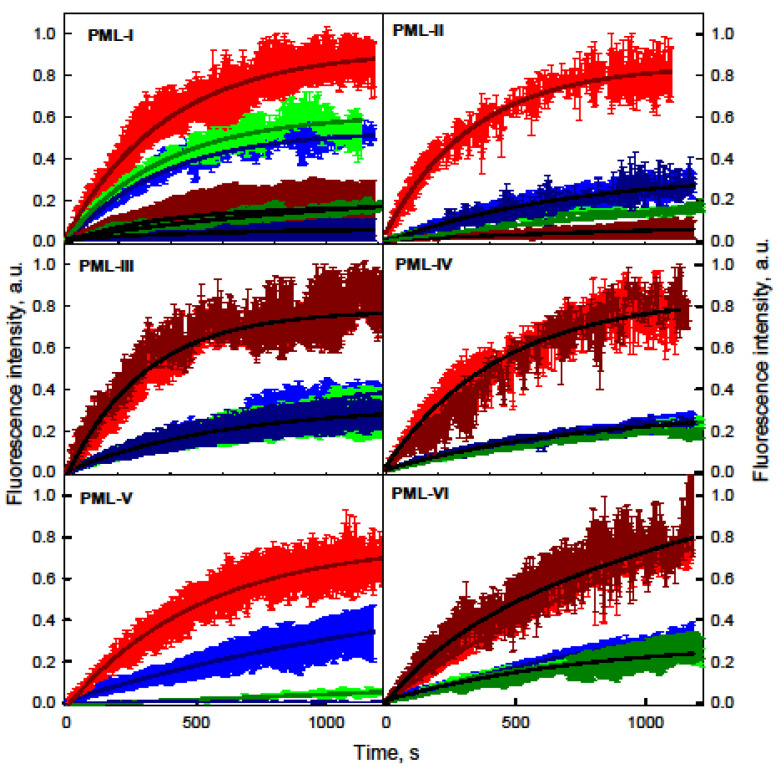
The acute oxidative stress action on the dynamics of exchange of PML isoforms with nucleoplasm in U2OS cells. The red, blue, and green curves are similar to those in Figure 4. Dark red indicates the curves of photoreduction of PML isoforms in the composition of “small” PML- bodies in the presence of 500 μM H_2_O_2_, dark blue indicates the curves of photoreduction of PML isoforms in the composition of “large” PML bodies in the presence of 500 μM H_2_O_2_, dark green—curves of photoreduction of PML isoforms in APBs in the presence of 500 μM H_2_O_2_. Solid curves represent the approximation of FRAP data in the framework of the mono-exponential approximation. The standard deviation of the data is passed by error bars of the corresponding color.

Considering that all nuclear isoforms of PML contain conserved N-terminal cysteine residues and the sumoylation site K490, H_2_O_2_-induced oxidative stress should have the same effect on the dynamics of exchange of PML isoforms with nucleoplasm. However, treatment of cells with hydrogen peroxide did not lead to solidification of PML bodies and APBs but caused the complete immobilization of PML-V and incorporation of PML-I and PML-II into the scaffold proteins of these organelles (Figure 5).

The dynamics of the exchange of PML-III, PML-IV, PML-VI isoforms with nucleoplasm remained the same and did not lead to a change in the number of PML-body populations with different dynamics of these PML isoforms with nucleoplasm. Peroxide treatment of U2OS cells causes a slight increase in the size of APBs. The exchange dynamics of PML isoforms included in APBs under oxidative stress changed similarly to PML-bodies not colocalized with TRF1 (Figure 4 and Figure 5). As it was shown [64], three factors are required for the formation of intermolecular disulfide bonds:

(1) The spatial accessibility/physical proximity of the partner cysteine residues forming the disulfide bond.
(2) The difference between the pKa of the involved thiol groups and the pH of the local environment (with lower pH limiting reactivity and higher pH favoring increased reactivity).
(3) The redox environment (with less reactivity under more reducing conditions and greater reactivity under more oxidizing conditions).

The C-terminal domain of PML-V that contains α–helical motif is known to have a strong tendency to form hyperstable oligomers [46]. It is obvious that the low exchange dynamics with the nucleoplasm of the PML-V isoform in the absence of stress defines the long lifetime of this protein in a condensed state, which contributes to the formation of disulfide bonds by it under conditions of an increase in the concentration of reactive oxygen species. In the PML bodies, PML isoforms that rapidly exchange with nucleoplasm are not under such conditions. Therefore, the formation of disulfide bonds by them is difficult even under conditions of a high concentration of reactive oxygen species. The slowdown in the dynamics of the exchange of PML-I and PML-II isoforms under oxidative stress may be due to the formation of intermolecular disulfide bonds by the C-terminal regions of these proteins under conditions of spatial convergence of protein chains due to weak nonspecific interactions of their C-terminal domains since only these isoforms contain cysteine residues in these domains.

Therefore, data reported in this study provide strong evidence that, for the formation of disulfide bonds between PML molecules and their subsequent formation of an insoluble scaffold, specific conditions are necessary, which can only be provided by pre-assembled condensates of these proteins.

## 3. Materials and Methods

### 3.1. Plasmids

On the basis of the pEGFP-C1/3 vectors, plasmids carrying genes encoding fusion proteins of 7 PML isoforms with green fluorescent protein were developed and created. The sequences of PML isoforms according to the Uniprot P-29590 database were used as reference sequences. The PML-I isoform was kindly provided by Prof. Roger Everett (UK). On the basis of this construct, plasmids were created carrying the genes encoding the remaining PML isoforms. The resulting construct was supplied by PCR with Q5 polymerase (NEB, Ipswich, MA, USA) with the corresponding primers. PCR products were precipitated with ethyl alcohol with ammonium acetate, dissolved in water, and treated with restriction enzymes. Restriction products were purified from agarose gel with Cleanup Mini kit (Evrogen, Moscow, Russia) and ligated to each other with T4 ligase (Thermo Fisher Scientific, Waltham, MA, USA).

Electroporation of Escherichia coli DH5 alpha cells was performed with the resulting ligase mixture. Several clones were selected for sequencing. Isolation of plasmids was carried out with Plasmid Miniprep kit (Evrogen, Russia). Next, the inserts of the obtained plasmids were sequenced with the preparation of samples using the BigDye Terminator v3.1 Cycle Sequencing Kit (Thermo Fisher Scientific, USA) and their subsequent analysis using the ABI PRISM 3500 (Applied Biosystems, Foster City, CA, USA). Clones in which the translated gene sequence coincides with the reference were selected. The transfection of the developed constructs into U2OS cells was carried out using the Effectene agent according to the manufacturer’s instructions.

### 3.2. MTT Assay

The day before the test, 100 μL of U2OS cells (2 × 10^4^ cells per well) were seeded into each inner well of a 96-well plate, and 100 μL of cell medium was added to the outer wells. The cells were incubated for a day at a temperature of +37 °C and air humidity of 5%. The cells were then incubated. in an environment with different concentrations of hydrogen peroxide. The incubation time varied from 10 min to 1 day. After the cells were incubated in hydrogen peroxide (SigmaAldrich, St. Louis, MO, USA), the medium was changed, followed by the introduction of 10 μL of 3- (4,5-dimethylthiazol-2-yl) -2,5-diphenyl tetrazolium bromide (MTT) (SigmaAldrich, USA) at a concentration of 5 mg/mL into each well. After 2 h of incubation of cells at a temperature of 37 °C and air humidity of 5% and the formation of formazan crystals from MTT, the cell medium was removed from the wells. Formazan crystals were dissolved in 100 μL DMSO (SigmaAldrich, USA). The analysis of the absorption of the obtained solutions at 595 nm using a plate photometer iMark (Bio-rad, Hercules, CA, USA) made it possible to determine the concentration of H_2_O_2_ leading to inhibition of the growth of the cell population.

### 3.3. Live Cell Imaging

U2OS cells were cultured in DMEM (GIBCO, Gaithersburg, MD, USA) medium containing 10% FBS (Thermo Fisher, Waltham, MA, USA), penicillin, streptomycin, and glutamate (Thermo Fisher, USA) at 37 °C with 5% CO_2_ in a humidified incubator.

Cell imaging was performed by planting U2OS cells on pre-treated with polylysine (SigmaAldrich, St. Louis, MO, USA) 35 mm glass Ibidy and Eppendorf plates. The constructs were visualized by irradiating the cells with a laser with a wavelength of 488 nm to excite EGFP fluorescence. Visualization of the cells nuclei was performed using a fluorescent dye DAPI by irradiating the cells with a laser with a wavelength of 405 nm to excite dye fluorescence. The resulting fluorescence was recorded using an Olympus FV3000 confocal microscope (60× Oil immersion objective, NA 1.42). Images were corrected for background signal and high-frequency noise using ImageJ software.

### 3.4. Fluorescence Recovery after Photobleaching (FRAP)

The analysis of the dynamics of PML isoforms in PML-bodies was carried out by restoring the EGFP fluorescence after photobleaching according to the following scheme: obtaining three images of the studied object before its photobleaching, photobleaching of target structures by irradiating them with a 488 nm laser with a power of 10 mW for 5 s, obtaining an image photo restoration of target objects for 20 min. FRAP curves were normalized on post-bleach fluorescence intensity. Images were analyzed using ImageJ software. EGFP fluorescence photoreduction curves for various PML isoforms were characterized within the mono-exponential approximation, the half-recovery time of EGFP fluorescence, the proportion of mobile and immobile fractions for each PML isoform was determined. Curves were analyzed using Prism 7 (GraphPad Software, USA).

### 3.5. Evaluation of Intrinsic Disorder Predisposition of Various PML Isoforms

Per-residue intrinsic disorder predisposition, seven alternatively spliced isoforms of human PML protein were analyzed using a DiSpi web crawler for the rapid prediction and comparison of protein disorder profiles. This web-crawler was designed to aggregate the results from a number of well-known disorder predictors: PONDR^®^ VLXT [65], PONDR^®^ VL3 [66], PONDR^®^ VLS2B [67], PONDR^®^ FIT [68], IUPred2 (Short), and IUPred2 (Long) [69,70]. This tool enables the rapid generation of disorder profile plots for individual polypeptides as well as arrays of polypeptides. The prediction of LLPS PML-isoforms was performed using by PSPredictor software package [53].

## 4. Conclusions

Taken together, our data create the prerequisites for revising the currently accepted model of PML body biogenesis, according to which the formation of PML bodies is initiated by the oligomerization of PML isoforms that form an insoluble scaffold, to which, due to polyvalent, primarily SUMO/SIM interactions, client proteins are attracted, thereby forming a dynamic layer that exchanges its content with the environment.

We found a population of “small” PML bodies of spherical topology with high exchange dynamics of PML isoforms with nucleoplasm and a low proportion of immobilized proteins, which suggests their liquid state unrelated to the multivalent SUM/SIM interactions. Such structures can act as “seeds” or “embryos” of functionally active PML bodies, providing the necessary concentration of PML isoforms to attract client proteins and, in particular, enzymes that provide SUMOylation of PML molecules, as well as, possibly, the formation of intermolecular disulfide bonds between PML monomers. FRAP analysis of larger bodies with toroidal topology showed the existence of the insoluble scaffold in the structure of such organelles.

## Supplementary Materials

The following are available online at https://www.mdpi.com/article/10.3390/ijms22115821/s1, Figure S1: Intrinsic disorder predisposition of different alternatively spliced isoforms of human PML protein. Intrinsic disorder profile were generated by DiSpi web. (A) Canonical isoform PML-I (UniProt ID P29590-1; 882 residues). (B) PML-II (829 residues; region 571-882 (SSRELDDSSS...GLAERASQQS) is changed to CMEPMETAEP...PVPGARQAGL). (C) PML-III (641 residues; region 642-882 is missing, region 571-641: SSRELDDSSS...RESKFRVVIQ → VSSSPQSEVL...PPSLASPPAR). (D) PML-IV (633 residues; region 621-633: TQKISQLAAVNRE → SGFSWGYPHPFLI; region 634-882 is missing). (E) PML-V (611 residues; region 571-611: SSRELDDSSS...DPQAEDRPLV → VSGPEVQPRT...LRLGNFPVRH, region 612-882 is missing). (F) PML-VI (560 residues; region 553-560: EERVVVIS → GRERNALW, region 561-882 is missing). (G) PML-VII (435 residues, region 436-882 is missing, region 419-435: PEEAERVKAQVQALGLA → LPPPAHALTGPAQSSTH). In these analyses, regions with disorder scores above the 0.5 threshold are considered intrinsically disordered, whereas regions with the disorder scores between 0.25 and 0.5 are considered as flexible, Figure S2. PML-bodies associated with alternative telomere lengthening (APBs). Panel A. Image of APBs with co-expressed by EGFP-PML-III and TagRFP-TRF1 using confocal fluorescence microscopy. Presented by APB in four angles. Panel B. 3D-structure of APBs with co-expressed by EGFP-PML-III and TagRFP-TRF1 using confocal fluorescence microscopy (Vidio). Panel C. Size distribution of PML-bodies colocalized with TRF1 in U2OS cells. Panel D. Visualization using fluorescence confocal microscopy of EGFP and TagRFP of the distribution of PML and TRF1 with coexpression of EGFP-PML-III and TagRFP-TRFP1 in U2OS cells. Figure S3. The acute oxidative stress action on the dynamics of exchange of PML-VII with cytoplasm in U2OS cells. The blue/dark blue curves indicate the curves of photoreduction of PML-VII in the composition of "cytoplasmic PML compartments in the absence/presence of 500 μM H_2_O_2_. Solid curves represent the approximation of FRAP data in the framework of the mono-exponential approximation. The standard deviation of the data is passed by error bars of the corresponding color.
