# Supplementary material for: The Role of Non-Specific Interactions in Canonical and ALT-Associated PML-Bodies Formation and Dynamics
Source: Int J Mol Sci. 2021 May 29;22(11):5821. doi: 10.3390/ijms22115821 (PMC8198325; doi:10.3390/ijms22115821)
Supplement: Supplementary file 1 [file ijms-22-05821-s001.zip › Fonin Supplement_25-05-2021.docx]

The role of non-specific interactions in canonical and ALT-associated PML-bodies formation and dynamics

Alexander V. Fonin ^1,^*^,†^, Sergey A. Silonov ^1,†^, Olesya G. Shpironok ^1,2^, Iuliia A. Antifeeva ^1^, Alexey V. Petukhov ^3^, Anna E. Romanovich ^4^, Irina M. Kuznetsova ^1^, Vladimir N. Uversky ^5,^* and Konstantin K. Turoverov ^1,6,^*

^1^ Laboratory of Structural Dynamics, Stability and Folding of Proteins, Institute of Cytology, Russian
Academy of Sciences, 4 Tikhoretsky Ave., 194064 St. Petersburg, Russian Federation; silonovsa25@yandex.ru (S.A.S.); olesyashpironok@gmail.com (O.G.S.); julgag@yandex.ru (I.A.A.); imk@incras.ru (I.M.K.)

^2^ Research Center for Molecular Mechanisms of Aging and Age-Related Diseases, Moscow Institute of Physics and Technology, 141700 Dolgoprudny, Russian Federation; olesyashpironok@gmail.com

^3^ Almazov National Medical Research Centre, Institute of Hematology, 197341 St. Petersburg, Russian Federation; petukhov_av@almazovcentre.ru

^4^ St-Petersburg State University Science Park, Resource Center of Molecular and Cell Technologies,
Universitetskaya nab. 7-9, 199034 St. Petersburg, Russian Federation; a.romanovich@spbu.ru

^5^ Department of Molecular Medicine and Byrd Alzheimer’s Research Institute, Morsani College of Medicine, University of South Florida, Tampa, FL 33612, USA

^6^ Peter the Great St.-Petersburg Polytechnic University, Polytechnicheskaya 29, 195251 St. Petersburg, Russian Federation

***** Correspondence: alexfonin@incras.ru (A.V.F.); vuversky@usf.edu (V.N.U.); kkt@incras.ru (K.K.T.);
Tel.: +7-812-2971957 (K.K.T.); Fax: +7-812-2970341(K.K.T.)

**†** These authors contributed equally to this work.


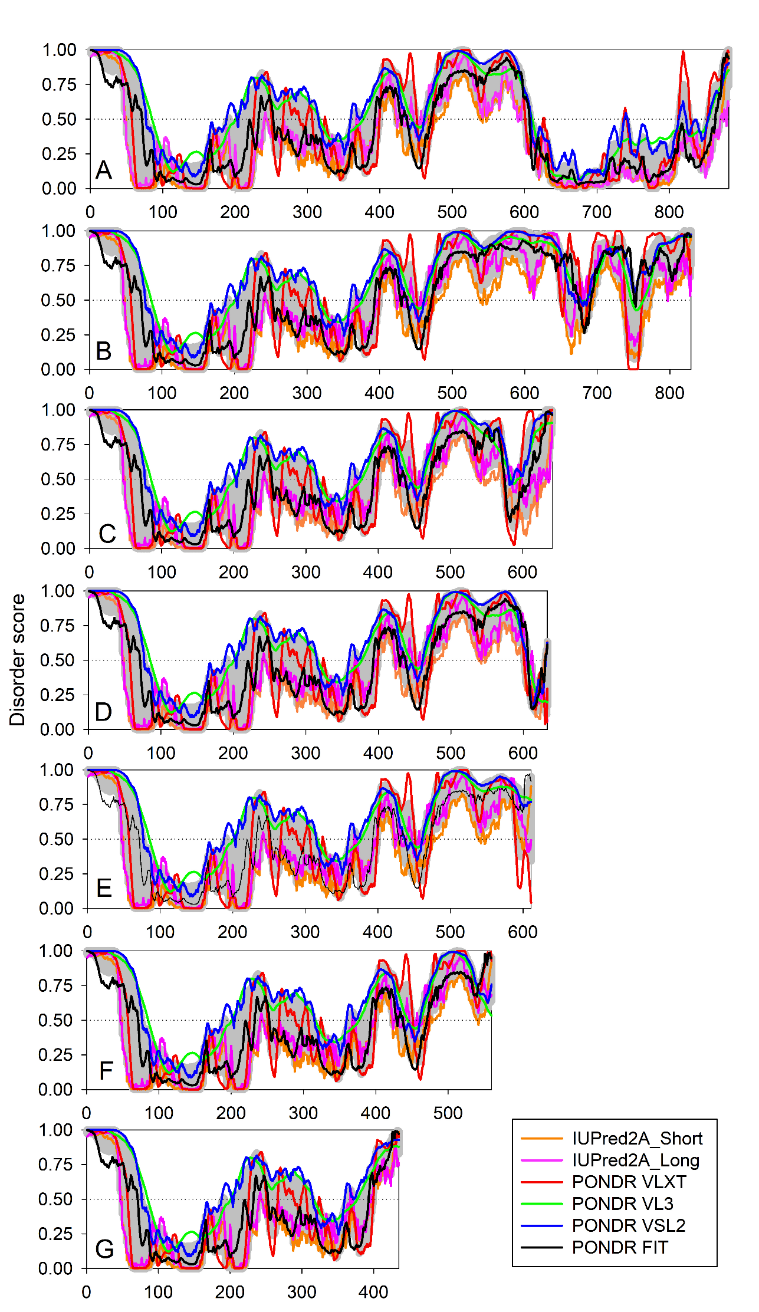


**Figure S1**. Intrinsic disorder predisposition of different alternatively spliced isoforms of human PML protein. Intrinsic disorder profile were generated by DiSpi web.

(A) Canonical isoform PML-I (UniProt ID P29590-1; 882 residues). (B) PML-II (829 residues; region 571-882 (SSRELDDSSS...GLAERASQQS) is changed to CMEPMETAEP...PVPGARQAGL). (C) PML-III (641 residues; region 642-882 is missing, region 571-641: SSRELDDSSS...RESKFRVVIQ → VSSSPQSEVL...PPSLASPPAR). (D) PML-IV (633 residues; region 621-633: TQKISQLAAVNRE → SGFSWGYPHPFLI; region 634-882 is missing). (E) PML-V (611 residues; region 571-611: SSRELDDSSS...DPQAEDRPLV → VSGPEVQPRT...LRLGNFPVRH, region 612-882 is missing). (F) PML-VI (560 residues; region 553-560: EERVVVIS → GRERNALW, region 561-882 is missing). (G) PML-VII (435 residues, region 436-882 is missing, region 419-435: PEEAERVKAQVQALGLA → LPPPAHALTGPAQSSTH). In these analyses, regions with disorder scores above the 0.5 threshold are considered intrinsically disordered, whereas regions with the disorder scores between 0.25 and 0.5 are considered as flexible.


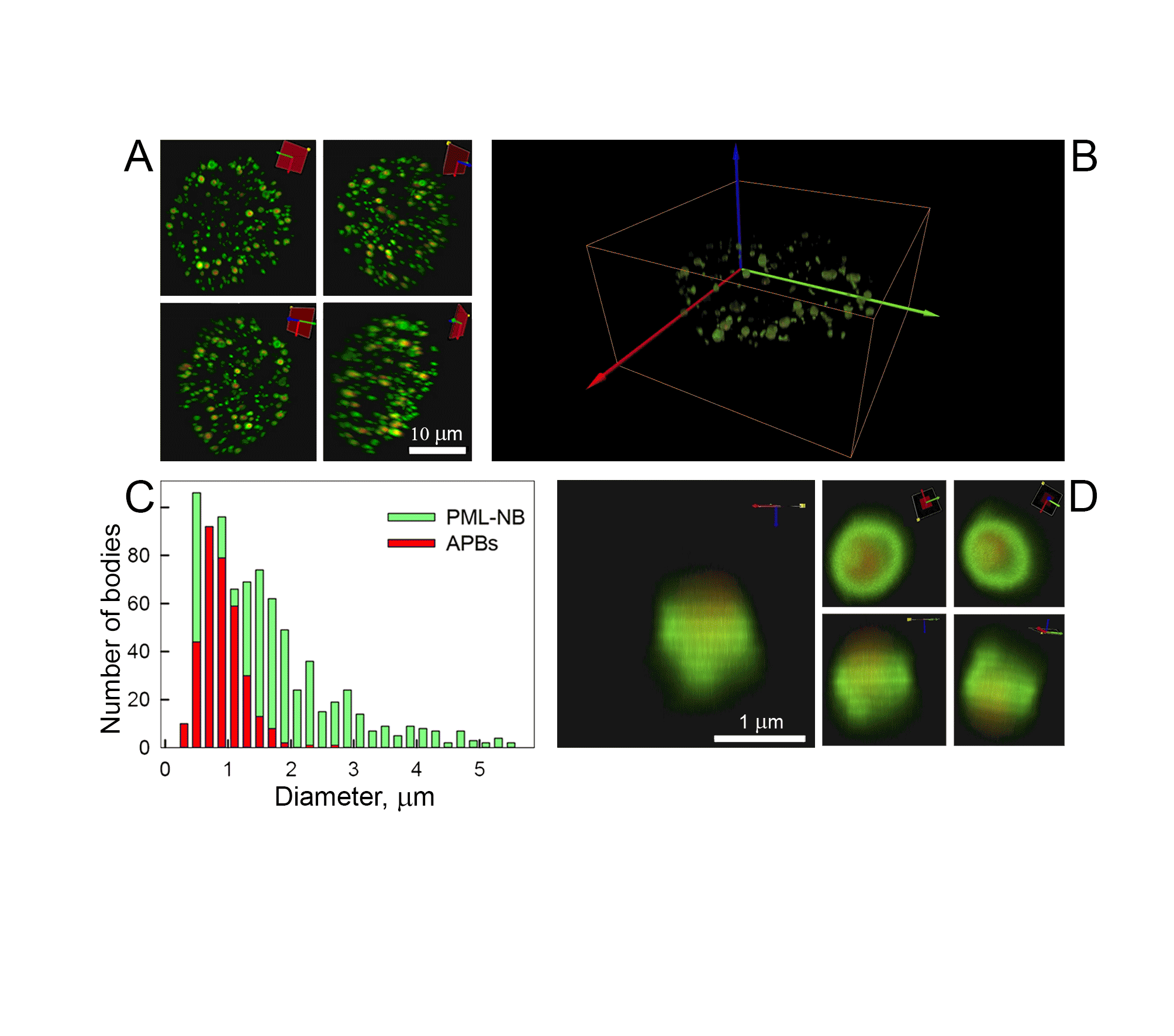


**Figure S2**. PML-bodies associated with alternative telomere lengthening (APBs).

Panel A. Image of APBs with co-expressed by EGFP-PML-III and TagRFP-TRF1 using confocal fluorescence microscopy. Presented by APB in four angles.

Panel B. 3D-structure of APBs with co-expressed by EGFP-PML-III and TagRFP-TRF1 using confocal fluorescence microscopy (Video).

Panel C. Size distribution of PML-bodies colocalized with TRF1 in U2OS cells.

Panel D. Visualization using fluorescence confocal microscopy of EGFP and TagRFP of the distribution of PML and TRF1 with coexpression of EGFP-PML-III and TagRFP-TRFP1 in U2OS cells.


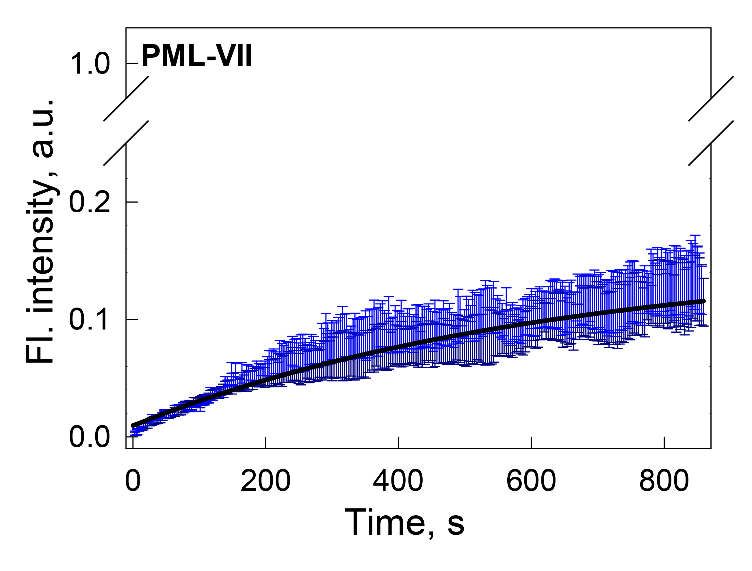


**Figure S3**. The acute oxidative stress action on the dynamics of exchange of PML-VII with cytoplasm in U2OS cells. The blue/dark blue curves indicate the curves of photoreduction of PML-VII in the composition of "cytoplasmic PML compartments in the absence/presence of 500 μM H_2_O_2_. Solid curves represent the approximation of FRAP data in the framework of the mono-exponential approximation. The standard deviation of the data is passed by error bars of the corresponding color.
