# Supplementary figures and images for: The Role of Non-Specific Interactions in Canonical and ALT-Associated PML-Bodies Formation and Dynamics
Source: Int J Mol Sci. 2021 May 29;22(11):5821. doi: 10.3390/ijms22115821 (PMC8198325; doi:10.3390/ijms22115821)

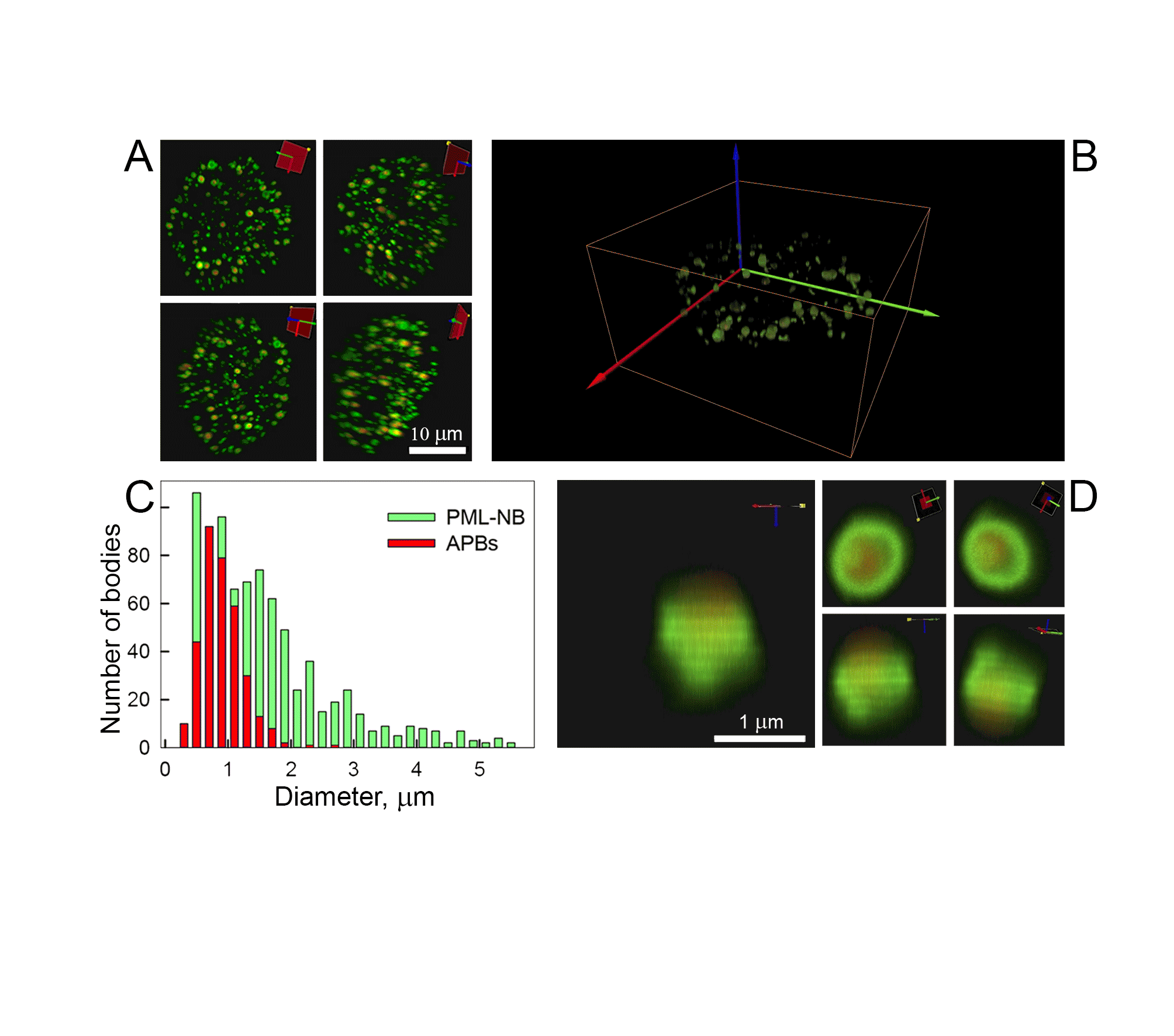

Supplement: Supplementary file 1 [file ijms-22-05821-s001.zip › Figure S2.gif]
